# Cathepsin L potentiates autoimmunity by inhibiting lysosome-mediated STING degradation

**DOI:** 10.3389/fimmu.2026.1920873

**Published:** 2026-08-26

**Authors:** Jia-Qing Xing, Zhi-Hao Zhang, Zeng-Lin Guo, Hong Cai, Qiu-Ying Han, Jie Pan, Min-Yi Feng, Wen Xue, Ming Zhao, Kai Wang, Xin Xu, Tao Li, Tian Xia, Li-Ming Sun

**Affiliations:** 1 Nanhu Laboratory, State Key Laboratory of Biomedical Analysis (SKLBA), Beijing, China; 2 Institute of Translational Medicine, Zhejiang University, Hangzhou, China

**Keywords:** AP1B1, cathepsin L, STING homeostasis, systemic lupus erythematosus, type I interferon

## Abstract

The stimulator of interferon genes (STING) orchestrates type I interferon (IFN) production in response to cytosolic DNA and plays essential roles in antiviral defense and autoimmune pathogenesis. The stability of STING determines the activation intensity of the pathway. Therefore, identifying proteins that govern its homeostatic regulation is needed. Here, we uncover the lysosomal protease cathepsin L (CTSL) as a critical stabilizer of STING in cells. CTSL deficiency selectively impairs STING-induced IFN responses without compromising overall lysosomal digestive function. Mechanistically, CTSL interacts with AP1B1 to prevent AP1B1-mediated lysosomal degradation of STING, thereby enhancing IFN signaling. Furthermore, *CTSL* expression is elevated in cells from systemic lupus erythematosus (SLE) patients and positively correlates with disease activity. Together, our findings establish an important role of CTSL in innate immunity by regulating STING homeostasis.

## 1 Introduction

The cGAS-STING pathway enables the host to mount type I interferon (IFN) responses against pathogen- and self-derived DNA (1–3). Cytosolic DNA is sensed by cyclic GMP-AMP synthase (cGAS), which catalyzes the production of the second messenger 2’3’-cyclic GMP-AMP (2’3’-cGAMP). 2’3’-cGAMP subsequently activates stimulator of interferon genes (STING, also known as MITA, MPYS or ERIS), an endoplasmic reticulum (ER)-localized adaptor protein. Activated STING recruits TANK-binding kinase 1 (TBK1), which phosphorylates STING and interferon regulatory factor 3 (IRF3) to induce the expression of type I IFN (4, 5). The intensity and duration of type I IFN responses must be tightly controlled, as insufficient activation permits viral escape, whereas excessive activation drives autoimmune diseases such as systemic lupus erythematosus (SLE) (3, 6–9).

To achieve precise control of type I IFN responses, activated STING is ultimately targeted for lysosome-dependent degradation to terminate signaling (10–12). Recent studies have established that adaptor protein complex 1 (AP-1) traffics activated STING to the lysosomal system for degradation, and disruption of AP-1 potentiates STING signaling (13). Consistently, pharmacological blockade of lysosome-dependent STING degradation potentiates type I IFN responses, thereby enhancing anti-tumor immunity (10). In addition, post-translational modifications critically regulate STING degradation. The E3 ubiquitin ligase RNF5 catalyzes K48-linked polyubiquitination of STING to promote its proteasomal degradation (14), while TRIM29 and TRIM30α similarly target STING for ubiquitin-dependent turnover (15, 16). Therefore, identifying proteins that stabilize STING against degradation is an attractive strategy to modulate type I IFN responses. TOLLIP was recently identified as one such stabilizer that directly binds STING and prevents its IRE1α-lysosome-mediated degradation in the resting cells (17). Yet additional STING stabilizers acting through distinct mechanisms have remained elusive.

Lysosomes are the cell’s primary degradative organelles, in which cathepsins constitute the major proteolytic enzymes (18, 19). Beyond their canonical degradative functions, lysosomes have emerged as signaling platforms in innate immunity. For example, lysosomal Toll-like receptors (TLRs) recognize pathogen-associated molecular patterns (PAMPs) to initiate type I IFN responses (20). The lysosomal membrane-anchored LAMTOR-Rag complex acts as a signaling center that regulates IRF3 and type I IFN responses (21). Nevertheless, whether hydrolases, particularly cathepsins, are involved in STING-dependent IFN regulation remains unclear.

Here, we show that CTSL deficiency impairs STING-dependent IFN responses by reducing STING protein levels without disrupting global lysosomal activity. CTSL physically interacts with AP1B1 and prevents it from mediating lysosomal degradation of STING, thereby acting as a selective stabilizer of STING. In patient samples, *CTSL* is upregulated in SLE patients and correlates with disease activity, highlighting its potential as a therapeutic target for STING-driven autoimmune diseases.

## 2 Materials and methods

### 2.1 Animals

C57BL/6JCya*-Ctsl^em1flox^* and C57BL/6JCya-Tg (EIIa-Cre) mice were purchased from Cyagen Biosciences. Wild-type C57BL/6 mice were from Charles River Laboratories. All animals were maintained under specific pathogen-free conditions with controlled temperature of 18-23 °C and 40-60% humidity under a 12 h light/12 h dark cycle.

### 2.2 Antibodies

Antibodies against cathepsin L (sc-390367) and β-Adaptin (AP1B1, sc-74423) were purchased from Santa Cruz Biotechnology. Antibodies against IRF3 (ab68481) and Goat Anti-Mouse IgG2a heavy chain (HRP, ab97245) were from Abcam. Antibodies against STING (19851-1-AP) and α-Tubulin (66031-1-Ig) were from Proteintech Group. Antibodies against p-STING (72971s), p-IRF3 (4947s) and cGAS (31659s) were from Cell Signaling Technology. Antibody against TREX1 (611986) was from BD Transduction Laboratories. Antibody against Flag (F3165) was from Sigma-Aldrich. All commercial antibodies have been verified by the manufacturers.

### 2.3 Reagents

HT-DNA (D6986) was purchased from Sigma-Aldrich. 2’3’-cGAMP (tlrl-nacga23-1), 3’3’-cGAMP (tlrl-nacga-1), c-di-AMP (tlrl-nacda), c-di-GMP (tlrl-nacdg) and poly(I:C) (tlrl-pic) were from InvivoGen. MG132 (S2619) was purchased from Selleckchem. Bafilomycin A1 (HY-100558), DQ-BSA-RED (HY-D2449), MSA-2 (HY-136927) and Z-FY-CHO (HY-128140) were from MedChemExpress. Lyso-Tracker Red (C1046) was from Beyotime Biotechnology. Anti-Flag Tag Magnetic Beads (BDAD0002) were from Biodragon.

### 2.4 Cell culture and treatment

L929 (CCL-1, ATCC) cells were cultured in MEM supplemented with 10% Fetal Bovine Serum (FBS), sodium pyruvate (1 mM), penicillin (100 U/mL) and streptomycin (100 μg/mL). Cells were cultured at 37 °C with 5% CO_2_. All cells used in the study were confirmed to be mycoplasma-free before use. Transfection of HT-DNA or poly(I:C) (2 μg/mL) was performed with Lipofectamine 2000 (Invitrogen). Cyclic dinucleotide stimulation was performed as previously described (4). Briefly, cells were incubated at 37 °C with 2’3’-cGAMP (1 μg/mL), 3’3’-cGAMP (2 μg/mL), c-di-AMP (20 μg/mL) and c-di-GMP (20 μg/mL) in permeabilization buffer (50 mM HEPES, pH 7.0; 100 mM KCl; 3 mM MgCl_2_; 0.1 mM DTT; 85 mM sucrose; 0.2% BSA; 1 mM ATP and 0.1 mM GTP) with 1 μg/mL digitonin (D141, Sigma). After incubation in permeabilization buffer for 30 min, the buffer was replaced with fresh MEM, and the cells were cultured for the indicated time. To assess the effect of Z-FY-CHO on *Ifnb* expression in L929 cells, cells were pretreated with Z-FY-CHO (20 µM) for 1 h, followed by stimulation with HT-DNA or other stimuli.

Primary peritoneal macrophages were isolated according to a previous publication (22). Briefly, 8-week-old mice were intraperitoneally (i.p.) injected with 4 mL of RPMI 1640 supplemented with 10% FBS, penicillin (100 U/mL) and streptomycin (100 μg/mL). After 1 min of gentle abdominal massage, the peritoneal lavage fluid was collected and centrifuged at 800 rpm for 3 min. The pelleted cells were resuspended and seeded into 12-well plates. The cells were then stimulated with HT-DNA (2 µg/mL) or 2’3’-cGAMP (1 µg/mL), and the expression of *Ifnb* was analyzed at the indicated time points.

### 2.5 HSV-1 infection

L929 cells or primary peritoneal macrophages were cultured in 12-well plates. Then, the cells were infected with HSV-1 (multiplicity of infection, MOI = 10). After incubation for the indicated times, the culture medium was removed, and cells were lysed with TRlzol reagent (T9424, Sigma-Aldrich) for further analysis.

### 2.6 ISD preparation

ISD was prepared from equimolar amounts of the sense and antisense DNA oligonucleotides (5’-TACAGATCTACTAGTGATCTATGACTGATCTGTACATGATCTACA-3’). Equimolar amounts of each strand were mixed and heated to 95 °C for 5 min in a thermal cycler and then slowly cooled to 25 °C at a rate of -1 °C/min to allow proper annealing. ISD was precipitated with 0.3 M sodium acetate (pH 5.2) and 2.5 volumes of pre-chilled absolute ethanol at -20 °C. After centrifugation, the pellet was washed with pre-chilled 70% ethanol, air-dried, and dissolved in endotoxin-free water. The obtained double-stranded DNA was verified by non-denaturing polyacrylamide gel electrophoresis and quantified spectrophotometrically (NanoDrop). Aliquots were stored at -20 °C until further use.

### 2.7 Flow cytometry

For Lyso-Tracker Red staining assay, the cells were pretreated with DMSO or BafA1 (0.5 μM) for 1 h, and then the cells were incubated with Lyso-Tracker Red (50 nM) for 10 min. Next, the cells were washed with buffer (PBS containing 1% FBS), and subjected to flow cytometry analysis using a CytoFLEX LX flow cytometer (Beckman Coulter). For each sample, the red lysosomal fluorescence of 10,000 cells was recorded and analyzed using FlowJo software.

### 2.8 Fluorescence microscopy

For live imaging to detect DQ-BSA-RED fluorescence dequenching, cells were seeded on 8-well chambered coverslips (C8-1.5P, Cellvis). Cells were pretreated with DMSO or BafA1 (0.5 μM) for 1 h. Then, cells were incubated with 10 μg/mL DQ-BSA-RED in culture medium for 4 h, followed by nuclear staining with 0.2 μg/mL Hoechst for 10 min prior to image acquisition. Images were acquired using a ZEISS LSM 900 (Zeiss) confocal microscope.

### 2.9 RNA extraction and RT-qPCR

Total RNA was isolated using TRIzol reagent according to the manufacturer’s instructions. The obtained RNAs were reverse-transcribed to cDNA using the PrimeScript RT Reagent Kit (RR037A, Takara). Then, the relative mRNA expression was analyzed using an Applied Biosystems QuantStudio 5 (Thermo Fisher Scientific) with PowerUp SYBR Green Master Mix (A25742, Thermo Fisher Scientific). Sequences of designed qPCR primers are listed in **Supplementary Table 1**.

### 2.10 Immunoblotting and immunoprecipitation

For immunoblotting, L929 cells and primary peritoneal macrophages were homogenized and lysed in RIPA lysis buffer (AR0102, BOSTE) supplemented with complete protease inhibitor cocktail. The lysed cells were vortexed and centrifuged at 12,000 × g for 15 min at 4 °C. The supernatant was then subjected to 8% - 12% SDS-PAGE and transferred to PVDF membranes. The membranes were blocked with 5% non-fat milk followed by incubation with the indicated primary antibodies overnight at 4 °C. After three washes with 1 × TBST, the membranes were probed with HRP-conjugated secondary antibodies, and the signals were detected using an Amersham ImageQuant 800 Western blot imaging system (Cytiva).

For immunoprecipitation, cells were lysed with NP-40 lysis buffer (AR0107, BOSTE) containing complete protease inhibitor cocktail, followed by centrifugation at 20,000 ×g for 15 min at 4 °C. The cell supernatants were immunoprecipitated with the indicated antibodies overnight at 4 °C and then incubated with agarose beads for another 4 h at 4 °C. For Flag immunoprecipitation, anti-Flag Tag Magnetic Beads were used for 2 h at 4 °C. The beads were washed four times with lysis buffer and then analyzed by immunoblotting.

### 2.11 Generation of stable cell lines

L929 knockout cell lines were generated with CRISPR/Cas9 technology. Briefly, lentiCRISPR v2 plasmid for targeting *Ctsl* was constructed. The guide RNA sequence (5’- CACCGGCTACCCATCAATTCACGAC-3’) was designed using the online tool CHOPCHOP (https://chopchop.cbu.uib.no/). HEK293T cells were transfected with plasmids (pVSVg, psPAX2 and sgRNA cloned lentiCRISPR v2 plasmid at a weight ratio of 1:3:4) using the enhanced calcium phosphate transfection kit (CTK001, MACGENE Biotechnology) and cultured for 48 h. Then the supernatant was collected and precipitated with PEG overnight at 4 °C. After a final centrifugation, the viral pellet was resuspended and added to L929 cells with polybrene (10 µg/mL) in 10-cm dishes for 48 h, followed by selection with puromycin (4 μg/mL). Protein expression was validated by immunoblotting. *Ctsl*^–/–^ cells were infected with lentivirus carrying the guide RNA-resistant wild-type *Ctsl* coding sequence to make rescue cell lines.

### 2.12 siRNA knockdown

A total of 30,000 L929 cells were transfected with siRNA using Lipofectamine RNAiMAX Transfection Reagent (13778075, Thermo Fisher Scientific). At 48 h post-transfection, the cells were stimulated with 2’3’-cGAMP for the indicated time points and *Ifnb* mRNA levels were analyzed as described above. Sequences of the designed siRNAs are listed in **Supplementary Table 2**.

### 2.13 Cell viability

L929 cells were seeded into 96-well plates at a density of 1×10^4^ cells per well and incubated with Z-FY-CHO at the indicated concentrations for 48 h. Cell viability was analyzed with the CellTiter 96 Aqueous One Solution Cell Proliferation Assay kit (G3580, Promega) according to the manufacturer’s instructions.

### 2.14 cGAMP extraction and measurement

L929 cells were stimulated with HT-DNA (2 μg/mL) for 2 h and then lysed with M-PER Mammalian Protein Extraction Reagent (78501, Thermo Fisher Scientific) for 15 min. After centrifugation at 15,000 rpm, the supernatant was collected and 2′3′-cGAMP levels in the cell lysates were measured using a 2′3′-cGAMP ELISA Kit (501700, Cayman Chemical) according to the manufacturer’s instructions. Finally, the absorbance was measured using a microplate reader at 450 nm.

### 2.15 Mass spectrometry

For sample preprocessing, proteins were reduced with dithiothreitol (DTT) and alkylated with iodoacetamide (IAM), followed by SP3 magnetic bead-based enrichment and digestion. Peptides were desalted using C18 Stage-Tip columns, lyophilized, and then subjected to Easy-nLC 1200/Q Exactive (Thermo Fisher Scientific). Each sample was subjected to a single LC-MS/MS run, generating a total of three raw MS files. All raw data were independently analyzed twice. MS raw data were searched against the customized UniProt database (uniprotkb_taxonomy_id_10090_AND_reviewe_2025_06_27.fasta) using MaxQuant (v2.4.9.0). Raw protein identification results were quality-controlled and preprocessed, and protein profiles were compared between the IgG control and CTSL immunoprecipitation groups. Proteins uniquely detected in the CTSL group (non-overlapping with the IgG group) were defined as CTSL-specific binding proteins and the corresponding proteins were selected for subsequent validation analysis. The MS analysis results are listed in **Supplementary Table 3**.

### 2.16 Bioinformatics analysis

To assess CTSL expression in SLE, multiple publicly available SLE datasets were retrieved from the Gene Expression Omnibus (GEO; https://www.ncbi.nlm.nih.gov/geo/). These datasets were generated using either RNA-seq or microarray platforms. For RNA-seq datasets, the reported transcripts per million (TPM) values were used to quantify CTSL expression. For microarray-based datasets, the provided normalized intensity values corresponding to CTSL probes were used for downstream analysis.

Data visualization was performed in R (v4.3.3) using ggplot2 (v3.5.1).

### 2.17 Data processing

The qPCR data were analyzed using QuantStudio Design & Analysis software. Fluorescence images were analyzed using ImageJ software. Densitometry quantification was performed with ImageJ software.

### 2.18 Statistical analysis

Statistical analyses were performed using GraphPad Prism 10. An unpaired two-tailed Student’s *t*-test or Spearman’s rank correlation was used for statistical analysis. Data are presented as the mean ± SD. For all analyses, statistical significance was defined as * *p* < 0.05, ** *p*<0.01, *** *p*<0.001, **** *p*<0.0001, NS, not significant.

## 3 Results

### 3.1 CTSL deficiency impairs cGAS-STING-mediated IFN signaling

**Figure 1:**
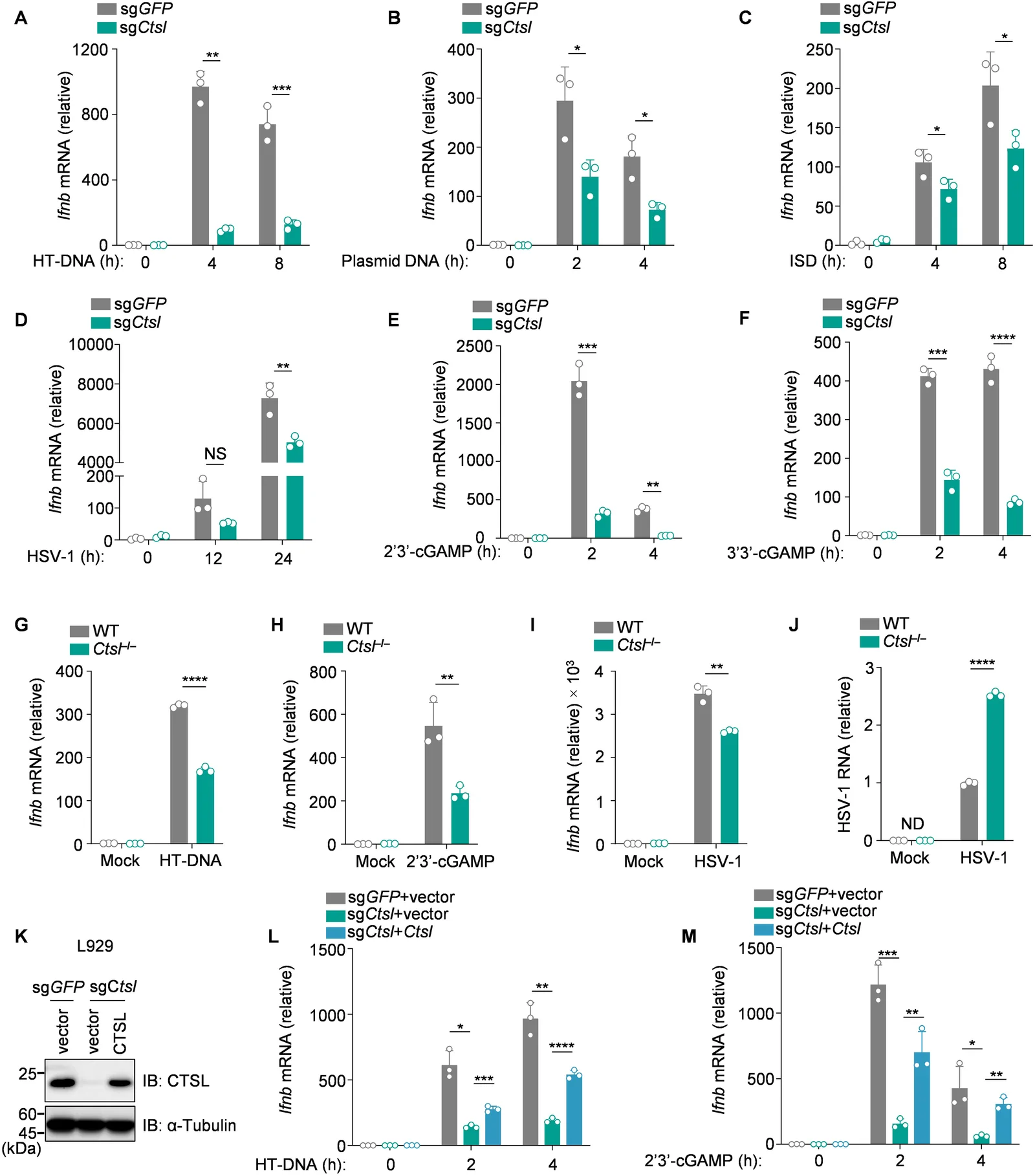
CTSL deficiency impairs cGAS-STING mediated IFN signaling. **(A-D)** qPCR analysis of *Ifnb* mRNA expression in L929 cells transfected with HT-DNA (2 μg/ml) **(A)**, plasmid DNA (2 μg/ml) **(B)**, ISD (2μg/ml) **(C)** or HSV-1 (MOI = 10) **(D)** for the indicated times. **(E, F)** qPCR analysis of *Ifnb* mRNA expression in L929 cells treated with 2’3’-cGAMP (1 μg/ml) **(E)** or 3’3’-cGAMP (2 μg/ml) **(F)**. **(G-I)** qPCR analysis of *Ifnb* mRNA expression in mouse primary peritoneal macrophages (PMs) transfected with HT-DNA (2 μg/ml) **(G)**, 2’3’-cGAMP (2 μg/ml) **(H)** or infected with HSV-1 (MOI = 10) **(I)**. **(J)** qPCR analysis of HSV-1 RNA in PMs that were untreated or infected with HSV-1 (MOI = 10). **(K)** Immunoblot analysis of the rescued proteins as shown. **(L, M)** qPCR analysis of *Ifnb* mRNA expression in the indicated rescued cells transfected with HT-DNA **(L)** or 2’3’-cGAMP **(M)** for the indicated times. Data are shown as mean ± SD of n = 3. *p < 0.05, **p < 0.01, ***p < 0.001, ****p < 0.0001. Statistical significance was determined using two-tailed unpaired Student’s t-test.

To investigate the role of CTSL in the cGAS-STING signaling pathway, we used the murine fibrosarcoma cell line L929, a well-established model for studying STING-dependent type I IFN responses (4). We deleted *Ctsl* in L929 cells using CRISPR/Cas9 (**Supplementary Figure 1A**) and examined the role of CTSL in cGAS-STING-mediated type I IFN production by measuring *Ifnb* expression. Compared with control cells, CTSL deficiency resulted in a marked reduction in *Ifnb* expression induced by various intracellular DNAs, including herring testis DNA (HT-DNA), plasmid DNA and interferon-stimulatory DNA (ISD, a 45-base-pair double-stranded DNA) (**Figures 1A–C**). The cGAS-STING pathway is critical for immune defense against DNA viruses such as herpes simplex virus-1 (HSV-1) (23). Consistently, HSV-1-induced *Ifnb* expression was also markedly reduced in *Ctsl*-deficient L929 cells compared with that in control cells (**Figure 1D**). CTSL deficiency also potently suppressed *Ifnb* expression induced by the STING ligands, including the endogenous ligand 2’3’-cGAMP and bacteria-derived cyclic dinucleotides such as c-di-AMP, c-di-GMP and 3’3’-cGAMP (**Figures 1E, F**, **Supplementary Figures 1B, C**). Consistent conclusions were further confirmed by using HT-DNA, 2’3’-cGAMP and HSV-1 in primary peritoneal macrophages from WT and *Ctsl^−/−^* mice (**Figures 1G–J**, **Supplementary Figures 1D, E**). To exclude the possible effect of CTSL on RNA signaling, we then challenged L929 cells with intracellular RNA mimic, poly(I:C), a synthetic analogue of dsRNA that activates intracellular RNA-sensing receptors. CTSL deficiency had no effect on poly(I:C)-mediated *Ifnb* expression (**Supplementary Figure 1F**).

To further validate the role of CTSL, we treated cells with Z-FY-CHO, a selective CTSL inhibitor, and found that it suppressed *Ifnb* expression induced by DNA or 2’3’-cGAMP (**Supplementary Figures 1G–J**). At this concentration, Z-FY-CHO had no obvious cytotoxic effect (**Supplementary Figure 1K**). Finally, we reconstituted wild-type CTSL in *Ctsl*-deficient L929 cells (**Figure 1K**), and found that stable expression of CTSL partially restored *Ifnb* expression (**Figures 1L, M**). Taken together, these data suggest that CTSL is critically required for cGAS-STING-mediated type I IFN production.

### 3.2 *Ctsl* knockout leads to the degradation of STING

**Figure 2:**
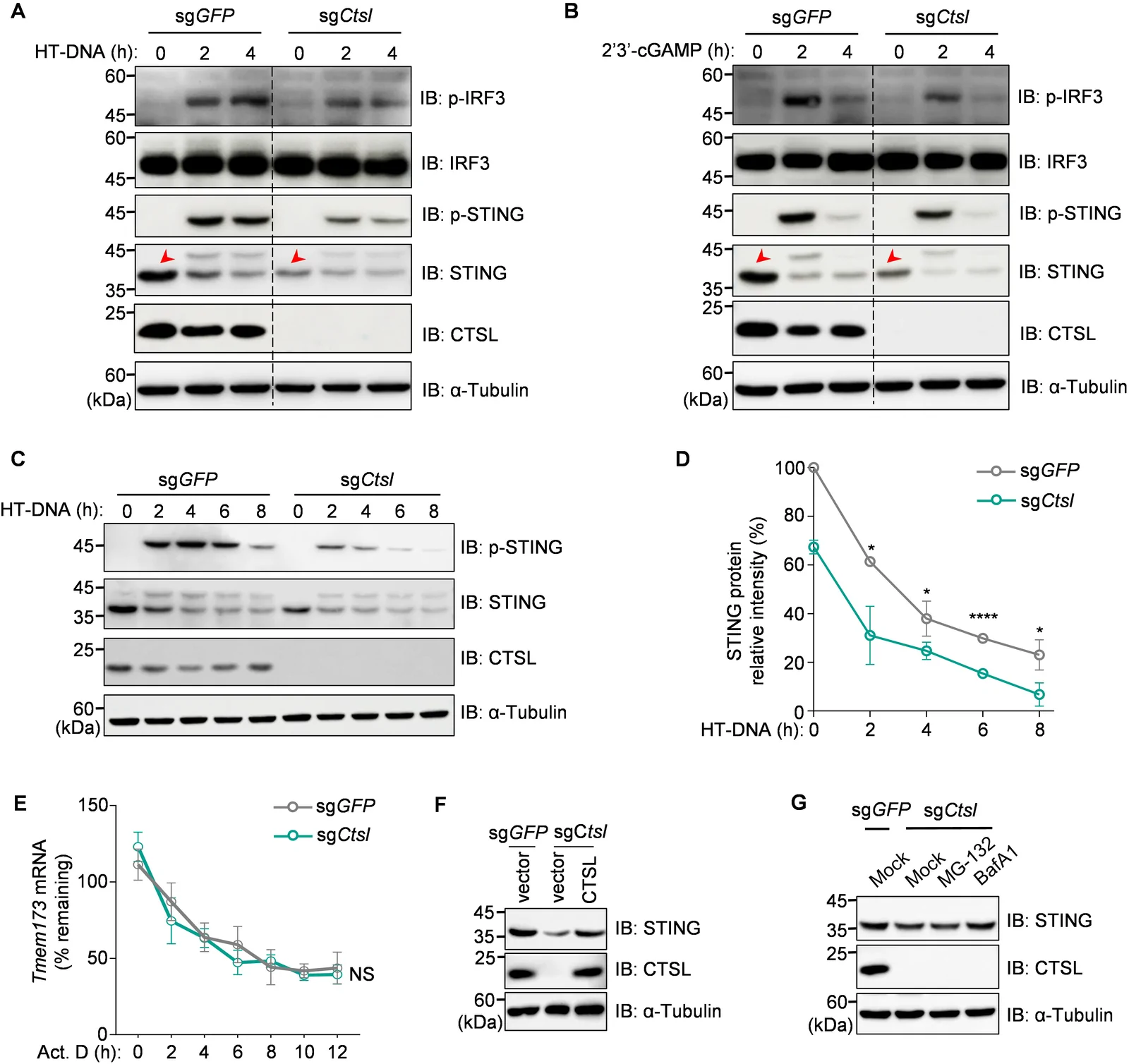
*Ctsl* knockout leads to the degradation of STING. **(A, B)** Immunoblot analysis of the indicated proteins in L929 cells stimulated with HT-DNA (2 μg/ml) **(A)** or 2’3’-cGAMP **(B)**. **(C)** Immunoblot analysis of control or *Ctsl*-deficient L929 cells stimulated with HT-DNA (2 μg/ml) over a time course. **(D)** Quantification of relative intensities of STING protein bands in **(C)**. **(E)** qPCR analysis of *Tmem173* mRNA turnover after actinomycin D (Act. D) treatment. **(F)** Immunoblot analysis of the indicated proteins in control and *Ctsl*-deficient L929 cells stably expressing an empty vector or *Ctsl* coding sequence. **(G)** Immunoblot analysis of control or *Ctsl*-deficient L929 cells treated overnight with MG-132 (5 μM) or BafA1 (0.5 μM). Data are shown as mean ± SD of n = 3. NS, not significant, *p < 0.05, ****p < 0.0001. Statistical significance was determined using two-tailed unpaired Student’s t-test.

To pinpoint the step of the cGAS-STING pathway that is affected by CTSL, we first examined the effect of CTSL on DNA-induced 2’3’-cGAMP synthesis. 2’3’-cGAMP synthesis was not significantly altered in *Ctsl*-deficient L929 cells, and cGAS protein levels were also comparable between the two genotypes (**Supplementary Figures 2A, B**), indicating that CTSL acts downstream of cGAS. Phosphorylation of STING and IRF3 is a critical hallmark of cGAS-STING pathway activation (4, 5). Upon HT-DNA stimulation, *Ctsl*-deficient L929 cells showed reduced IRF3 and STING phosphorylation (**Figure 2A**), and similar results were obtained with 2’3’-cGAMP stimulation (**Figure 2B**). Notably, total STING protein levels were substantially decreased even in resting *Ctsl*-deficient cells, whereas total IRF3 levels remained unchanged (**Figures 2A, B**, indicated by red arrows). To further characterize STING degradation following *Ctsl* deletion, we next performed a time-course analysis. HT-DNA-stimulated cells confirmed that STING protein levels were consistently lower in *Ctsl*-deficient cells than in controls at all time points examined (**Figures 2C, D**). To determine whether the reduction in STING protein resulted from changes at the RNA level, we measured *Tmem173* mRNA and found that neither steady-state levels (**Supplementary Figure 2C**) nor mRNA stability, as assessed by actinomycin D chase (**Figure 2E**), differed between control and *Ctsl*-deficient cells. Reconstitution of wild-type CTSL in *Ctsl*-deficient cells restored STING protein to control levels (**Figure 2F**). These data indicate that CTSL promotes the cGAS-STING signaling pathway by maintaining STING protein levels rather than by modulating STING transcription.

STING is known to be degraded via the lysosomal or proteasomal pathways after activation, a mechanism that prevents excessive interferon production (11–16). To explore which pathway mediates STING degradation in the absence of CTSL, we treated L929 cells with inhibitors of proteasomal degradation (MG-132) or lysosomal acidification (bafilomycin A1, BafA1). In *Ctsl*-deficient cells, BafA1 restored STING protein to levels comparable to those in control cells, whereas MG-132 treatment led to only a modest increase (**Figure 2G**). These results suggest that CTSL protects STING primarily from lysosomal degradation.

### 3.3 CTSL deficiency does not affect the function of lysosomes

**Figure 3:**
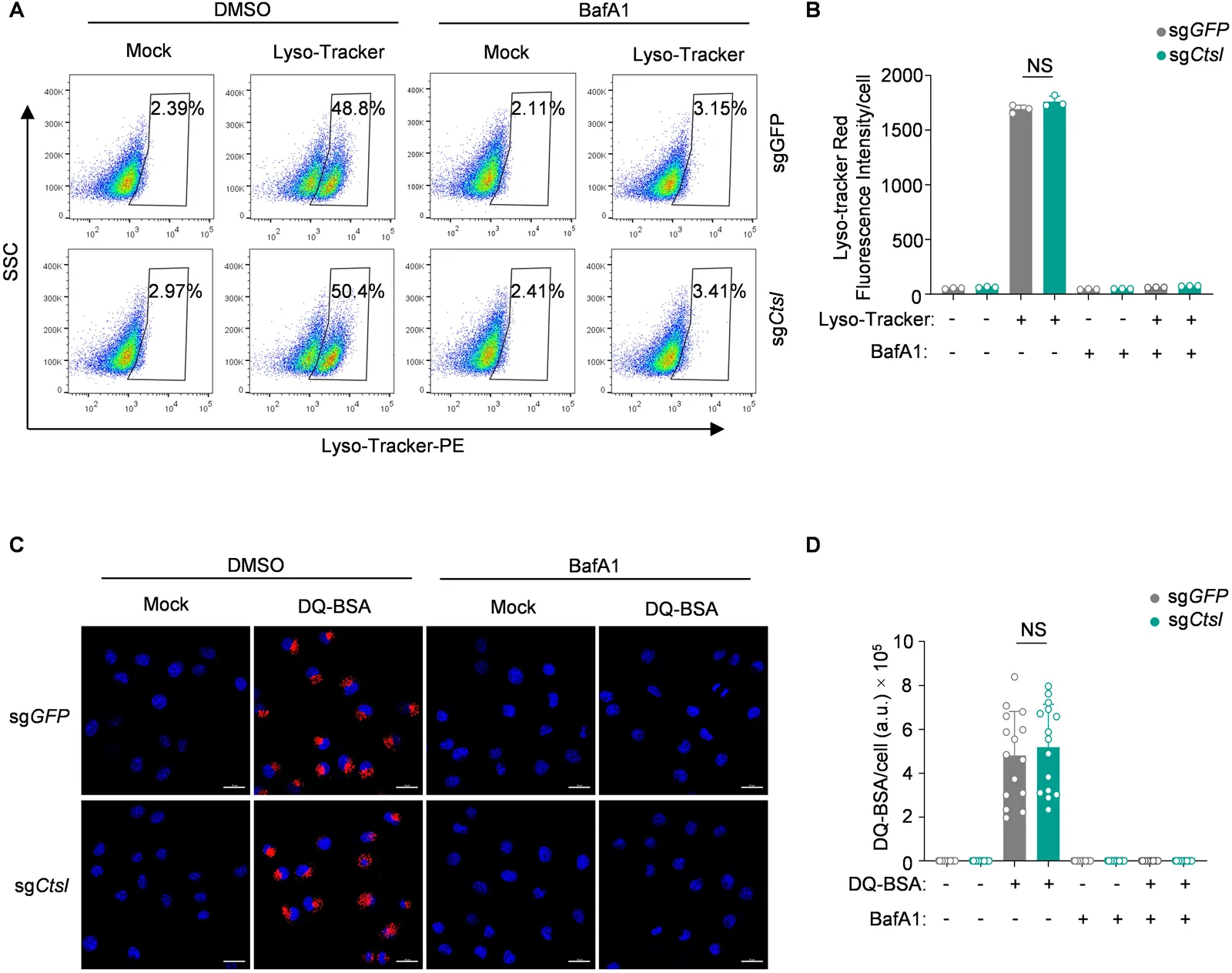
CTSL deficiency does not affect the function of lysosomes. **(A)** Flow cytometry analysis of L929 cells following staining with Lyso-Tracker. The BafA1-treated groups served as positive controls. **(B)** Quantification of Lyso-Tracker fluorescence intensity in cells shown in **(A)**. **(C)** Lysosomal DQ-BSA-RED degradation in L929 cells, analyzed by microscopy, with BafA1-treated groups as controls. Scale bars, 20 μm. **(D)** Quantification of DQ-BSA-RED degradation in cells shown in **(C)**. Data are shown as mean ± SD of n = 3 for **(B)** and n = 15 fields of view with ≥10 cells for **(D)**. NS, not significant. Statistical significance was determined using two-tailed unpaired Student’s t-test.

The observation that STING is degraded in the absence of CTSL raised the question of whether this reflects global lysosomal dysfunction. To rule out this possibility, we first used Lyso-Tracker, a fluorescent probe that accumulates in acidic compartments and serves as a marker of lysosomal acidification (24). Lyso-Tracker fluorescence was comparable between *Ctsl*-deficient and control cells, whereas BafA1 treatment markedly reduced the signal as expected (**Figures 3A, B**, **Supplementary Figure 3A**), indicating that *Ctsl* deficiency does not affect lysosomal acidification. To further assess lysosomal proteolytic activity, we employed another marker of lysosomal function, DQ-BSA-RED, which displays bright red fluorescence only upon proteolytic cleavage (25). Confocal imaging revealed that DQ-BSA fluorescence was comparable between *Ctsl*-deficient and control cells, but was strongly reduced by BafA1 treatment (**Figures 3C, D**). Collectively, these results exclude global lysosomal dysfunction and point to a specific, CTSL-dependent mechanism that selectively maintains STING stability, prompting us to identify the molecular partner that mediates this protection.

### 3.4 CTSL interacts with AP1B1 to control STING-mediated immune responses

**Figure 4:**
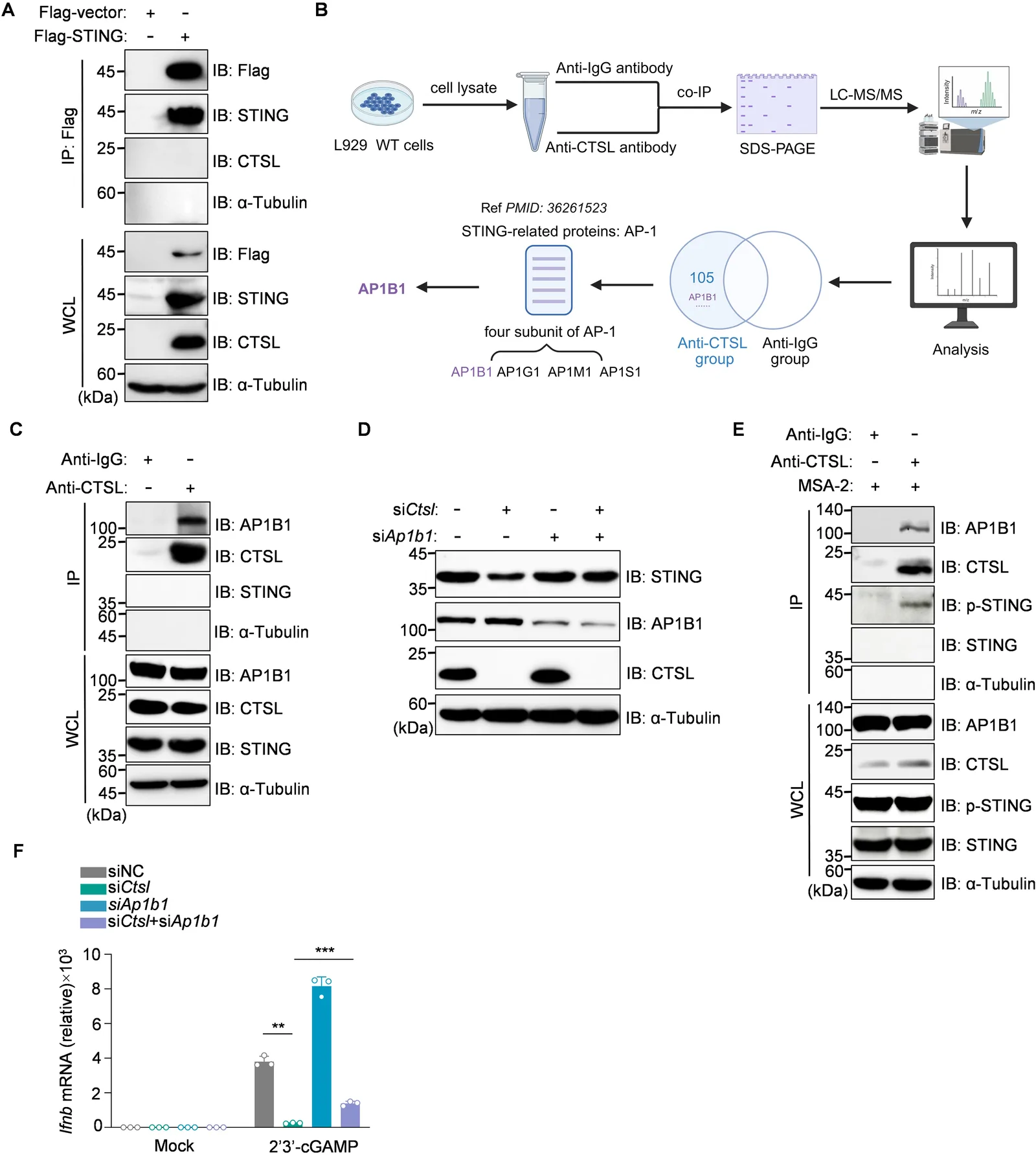
CTSL interacts with AP1B1 to control STING-mediated immune responses. **(A)** Immunoblot analysis of the indicated proteins following immunoprecipitation of Flag-STING. **(B)** Experimental design to identify candidate CTSL-interacting STING-related proteins. Created in BioRender. Sun, L. (2026) https://BioRender.com/bbw3j7e. **(C)** Co-IP of CTSL and AP1B1 was analyzed by immunoblotting. Anti-CTSL was used to pull down AP1B1 or STING. Anti-IgG served as a negative control. **(D)** Immunoblot analysis of the indicated proteins in L929 cells transfected with siRNAs. **(E)** L929 cells were stimulated with MSA-2. After co-IP with anti-CTSL or anti-IgG antibody, samples were analyzed by immunoblotting. **(F)** qPCR analysis of *Ifnb* mRNA expression in L929 cells transfected with the indicated siRNAs and then treated with 2’3’-cGAMP (1 µg/mL) for 2 h. Data are shown as mean ± SD of n = 3. **p < 0.01, ***p < 0.001. Statistical significance was determined using two-tailed unpaired Student’s t-test.

We first tested whether CTSL directly interacts with STING by co-immunoprecipitation (co-IP), but no interaction was detected between CTSL and STING (**Figure 4A**). To identify CTSL-interacting proteins, we next performed co-IP coupled with mass spectrometry on samples from L929 lysates and identified the potential STING-related proteins that specifically bind to CTSL as the targets (**Figure 4B**). Among the identified proteins was AP1B1, a subunit of the AP-1 complex, previously shown to mediate lysosomal degradation of STING (13). Reciprocal co-IP experiments confirmed the interaction between CTSL and AP1B1 in L929 cells (**Figure 4C**). We performed epistasis analysis by gene knockdown experiments to confirm AP1B1-mediated regulation of basal STING protein stability. Single knockdown of *Ctsl* resulted in a marked reduction in the levels of STING, whereas dual knockdown of *Ctsl* and *Ap1b1* completely restored STING protein levels (**Figure 4D**), confirming that AP1B1 mediates resting STING degradation in the absence of CTSL.

Although CTSL did not directly bind STING, we asked whether they reside in the same complex upon activation. Following stimulation with the STING agonist MSA-2 (26), phosphorylated STING (p-STING), but not total STING, colocalized with CTSL (**Figure 4E**). Accordingly, we assessed the functional role of the CTSL-AP1B1 complex in STING signaling. *Ctsl* knockdown markedly reduced *Ifnb* expression compared with control cells. In contrast, *Ap1b1* knockdown alone increased *Ifnb* expression (**Figure 4F**), consistent with previous work (13). Importantly, simultaneous knockdown of both *Ctsl* and *Ap1b1* largely restored the impaired *Ifnb* expression, indicating that *Ap1b1* depletion reversed the functional defect caused by CTSL deficiency (**Figure 4F**). Collectively, these results suggest that, by binding AP1B1, CTSL maintains STING protein stability and downstream signaling.

### 3.5 CTSL contributes to IFN-related autoimmune responses

**Figure 5:**
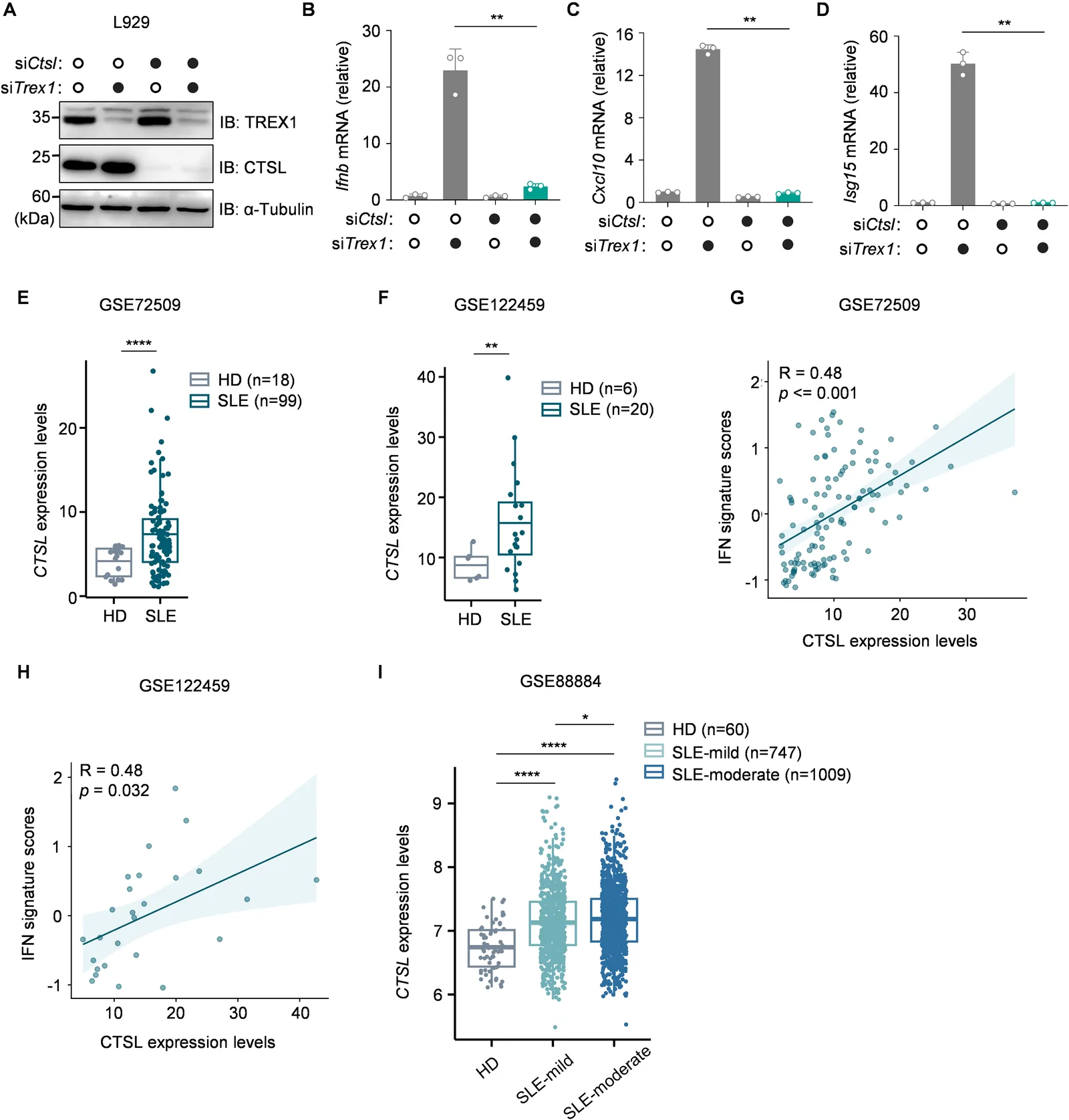
CTSL contributes to IFN-related autoimmune responses. **(A)** Immunoblot analysis of the indicated proteins in L929 cells treated with *Ctsl* or *Trex1* siRNA. **(B–D)** qPCR analysis of ISGs in L929 cells transfected with *Ctsl* or *Trex1* siRNAs. **(E, F)** Boxplots showing *CTSL* expression in SLE patients and healthy donors (HD) from PBMC-derived datasets (GSE72509 and GSE122459). **(G, H)** Association between *CTSL* expression levels and IFN signature scores. **(I)** Correlation between CTSL expression and disease severity in SLE patients (GSE88884). Data are shown as mean ± SD. *p < 0.05, **p < 0.01, ****p < 0.0001. Statistical significance was determined using two-tailed unpaired Student’s t-test for **(B–F, I)** or Spearman correlation for **(G, H)**.

Given that CTSL deficiency dampens STING-mediated IFN production in cells, we next asked whether CTSL modulates IFN-related responses in a cellular model of autoimmunity. To test this, we knocked down *Trex1* in L929 cells (**Figure 5A**), which induced aberrant activation of the cGAS-STING pathway (27). As expected, *Trex1* knockdown promoted the expression of interferon-stimulated genes (ISGs), and co-depletion of *Ctsl* significantly suppressed the induction of ISGs (**Figures 5B–D**).

SLE is a prototypical autoimmune disease characterized by high levels of IFN, which correlate with disease progression. To explore the clinical relevance of CTSL in SLE, we analyzed public transcriptomic datasets (GSE72509; GSE122459) from SLE patients and healthy donors (HD). In two independent cohorts, *CTSL* expression was significantly upregulated in SLE patients compared with healthy donors (**Figures 5E, F**). To further assess its association with IFN activity, we calculated IFN signature scores based on a published IFN signature gene set (28, 29). In two independent SLE patient cohorts, patients with higher *CTSL* expression displayed markedly increased IFN signature scores, supporting an association between elevated *CTSL* levels and amplified IFN activity in patients (**Figures 5G, H**). We further stratified SLE patients by disease severity and found that individuals with moderate disease activity exhibited markedly higher CTSL expression than those with mild disease activity (GSE88884, **Figure 5I**). Collectively, these analyses showed that elevated CTSL expression is associated with SLE disease activity, highlighting CTSL as a promising biomarker and potential therapeutic target in SLE.

## 4 Discussion

In this study, we identified CTSL as a critical positive regulator of the cGAS-STING pathway. CTSL deficiency reduced the basal protein level of STING and specifically inhibited STING-dependent type I IFN expression. Mechanistically, CTSL physically interacts with the AP-1 subunit AP1B1 and prevents it from mediating lysosomal degradation of STING, thereby safeguarding STING protein stability. Unlike the established STING stabilizer TOLLIP, which directly binds STING, CTSL did not detectably interact with STING, indicating that CTSL safeguards STING indirectly through AP1B1 rather than by directly shielding STING itself.

Our findings support a dynamic dual-state model. Under basal conditions, the CTSL-AP1B1 complex constitutively protects STING from lysosomal degradation. Upon STING activation, phosphorylated STING colocalizes with the CTSL-AP1B1 complex, suggesting that the complex may engage activated STING to regulate downstream signaling. Within this framework, CTSL promotes IFN production whereas AP1B1 restrains it. This dual functionality explains why co-depletion of AP1B1 restores both STING protein levels and IFN responses in the absence of CTSL.

Importantly, this work uncovers a non-canonical function of a lysosomal hydrolase in innate immunity. Although CTSL is traditionally viewed as a lysosomal protease that executes terminal protein degradation, its depletion does not impair general lysosomal hydrolytic capacity, indicating that its role in STING regulation is separable from its proteolytic activity. Our study therefore expands the functional repertoire of CTSL by revealing a role in controlling the stability of an innate immune sensor.

Our findings may have implications for IFN-driven autoimmune diseases. SLE is a type I interferonopathy in which elevated IFN drives disease progression (30–32). The clinical efficacy of anifrolumab, an anti-interferon-α receptor monoclonal antibody, in moderate-to-severe SLE further confirms that aberrant interferon signaling is a central pathogenic driver of SLE (33). Building on prior reports, CTSL may sustain IFN production in SLE via two complementary mechanisms. Firstly, as an IFN-inducible molecule in monocytes and macrophages (34), CTSL is upregulated in the chronic IFN milieu of SLE and in turn stabilizes STING to form a pathogenic positive feedback loop, consistent with our finding that CTSL depletion blunts ISG induction in *Trex1*-deficient cells. Secondly, CTSL mediates proteolytic activation of endosomal TLR7/9 (35–37), and its elevation may lower the activation threshold for self-nucleic acids, driving IFN-α production. Clinically, *CTSL* expression is elevated in SLE patient cells, and correlates with disease activity and IFN signature scores across two independent cohorts. Taken together, these cell-based and correlative clinical data suggest that CTSL may contribute to sustained IFN production in SLE.

Several CTSL-targeting strategies have reached clinical evaluation (ClinicalTrials.gov), although none has been tested in autoimmune diseases. The dual SARS-CoV-2 Mpro/CTSL inhibitor olgotrelvir has been evaluated in a completed phase 3 trial in patients with COVID-19, and the oral CTSL inhibitor SLV213 has been evaluated in early-phase clinical studies, demonstrating the clinical tractability of CTSL targeting. Notably, hydroxychloroquine, a first-line conventional immunosuppressant for SLE, was recently shown to directly bind CTSL and suppress its enzymatic activity (38), providing a potential mechanistic link between hydroxychloroquine treatment and CTSL regulation in SLE patients. Given that CTSL promotes IFN production by stabilizing STING in cell-based models, pharmacological inhibition of CTSL may offer a therapeutic strategy for SLE and other IFN-driven autoimmune diseases.

We also acknowledge several limitations. Firstly, our experiments were conducted exclusively in cell-based models, and *in vivo* evidence from animal models of autoimmunity is lacking. Secondly, it remains unclear how a lysosome-resident protease encounters STING and AP1B1, and whether the CTSL-AP1B1-STING axis operates at a specific membrane compartment, such as recycling endosomes or the trans-Golgi network. Thirdly, although we demonstrate that CTSL modulates IFN signaling and correlates with SLE disease activity, direct modulation of CTSL in primary SLE cells needs to be further evaluated.

In summary, using cell-based models, we identify the CTSL-AP1B1 axis as a regulatory mechanism of the cGAS-STING pathway that controls both resting-state stability and activation-state signaling of STING. This study unveils a novel paradigm in which a lysosomal protease functions as a gatekeeper of innate immune signaling, opening new avenues for the treatment of IFN-driven autoimmune diseases.

## Data Availability

The tandem mass spectrometry data presented in the study are publicly available. This data can be found here: https://ngdc.cncb.ac.cn/omix/release/OMIX019821. Accession number: OMIX019821.

## Author’s note

The State Key Laboratory of Biomedical Analysis (SKLBA) was formerly know as the National Center of Biomedical Analysis.

## Supplementary material

Supplementary Table 3 The MS analysis results.

## References

[1] Tan X Sun L Chen J Chen ZJ . Detection of microbial infections through innate immune sensing of nucleic acids. Annu Rev Microbiol. (2018) 72:447–78. doi: 10.1146/annurev-micro-102215-095605 30200854

[2] Zhang B Xu P Ablasser A . Regulation of the cGAS-STING pathway. Annu Rev Immunol. (2025) 43:667–92. doi: 10.1146/annurev-immunol-101721-032910 40085836

[3] Zhang Z Zhang C . Regulation of cGAS-STING signalling and its diversity of cellular outcomes. Nat Rev Immunol. (2025) 25:425–44. doi: 10.1038/s41577-024-01112-7 39774812

[4] Wu J Sun L Chen X Du F Shi H Chen C . Cyclic GMP-AMP is an endogenous second messenger in innate immune signaling by cytosolic DNA. Science. (2013) 339:826–30. doi: 10.1126/science.1229963 23258412 PMC3855410

[5] Sun L Wu J Du F Chen X Chen ZJ . Cyclic GMP-AMP synthase is a cytosolic DNA sensor that activates the type I interferon pathway. Science. (2013) 339:786–91. doi: 10.1126/science.1232458 23258413 PMC3863629

[6] Chen C Xu P . Cellular functions of cGAS-STING signaling. Trends Cell Biol. (2023) 33:630–48. doi: 10.1016/j.tcb.2022.11.001 36437149

[7] Ablasser A Chen ZJ . cGAS in action: Expanding roles in immunity and inflammation. Science. (2019) 363. doi: 10.1126/science.aat8657 30846571

[8] Motwani M Pesiridis S Fitzgerald KA . DNA sensing by the cGAS-STING pathway in health and disease. Nat Rev Genet. (2019) 20:657–74. doi: 10.1038/s41576-019-0151-1 31358977

[9] Dobbs N Burnaevskiy N Chen D Gonugunta VK Alto NM Yan N . STING activation by translocation from the ER is associated with infection and autoinflammatory disease. Cell Host Microbe. (2015) 18:157–68. doi: 10.1016/j.chom.2015.07.001 26235147 PMC4537353

[10] Gonugunta VK Sakai T Pokatayev V Yang K Wu J Dobbs N . Trafficking-mediated STING degradation requires sorting to acidified endolysosomes and can be targeted to enhance anti-tumor response. Cell Rep. (2017) 21:3234–42. doi: 10.1016/j.celrep.2017.11.061 29241549 PMC5905341

[11] Gentili M Liu B Papanastasiou M Dele-Oni D Schwartz MA Carlson RJ . ESCRT-dependent STING degradation inhibits steady-state and cGAMP-induced signalling. Nat Commun. (2023) 14:611. doi: 10.1038/s41467-023-36132-9 36739287 PMC9899276

[12] Pan M Yin Y Hu T Wang X Jia T Sun J . UXT attenuates the CGAS-STING1 signaling by targeting STING1 for autophagic degradation. Autophagy. (2023) 19:440–56. doi: 10.1080/15548627.2022.2076192 35543189 PMC9851252

[13] Liu Y Xu P Rivara S Liu C Ricci J Ren X . Clathrin-associated AP-1 controls termination of STING signalling. Nature. (2022) 610:761–7. doi: 10.1038/s41586-022-05354-0 36261523 PMC9605868

[14] Zhong B Zhang L Lei C Li Y Mao AP Yang Y . The ubiquitin ligase RNF5 regulates antiviral responses by mediating degradation of the adaptor protein MITA. Immunity. (2009) 30:397–407. doi: 10.1016/j.immuni.2009.01.008 19285439

[15] Wang Y Lian Q Yang B Yan S Zhou H He L . TRIM30alpha is a negative-feedback regulator of the intracellular DNA and DNA virus-triggered response by targeting STING. PloS Pathog. (2015) 11:e1005012. doi: 10.1371/journal.ppat.1005012 26114947 PMC4482643

[16] Li Q Lin L Tong Y Liu Y Mou J Wang X . TRIM29 negatively controls antiviral immune response through targeting STING for degradation. Cell Discov. (2018) 4:13. doi: 10.1038/s41421-018-0010-9 29581886 PMC5859251

[17] Pokatayev V Yang K Tu X Dobbs N Wu J Kalb RG . Homeostatic regulation of STING protein at the resting state by stabilizer TOLLIP. Nat Immunol. (2020) 21:158–67. doi: 10.1038/s41590-019-0569-9 31932809 PMC6983345

[18] Lim CY Zoncu R . The lysosome as a command-and-control center for cellular metabolism. J Cell Biol. (2016) 214:653–64. doi: 10.1083/jcb.201607005 27621362 PMC5021098

[19] Bonam SR Wang F Muller S . Lysosomes as a therapeutic target. Nat Rev Drug Discov. (2019) 18:923–48. doi: 10.1038/s41573-019-0036-1 31477883 PMC7097195

[20] Fitzgerald KA Kagan JC . Toll-like receptors and the control of immunity. Cell. (2020) 180:1044–66. doi: 10.1016/j.cell.2020.02.041 32164908 PMC9358771

[21] Feng Z Wang L Chen S Cao S Lei M Yang X . The lysosomal LAMTOR-Rag complex functions as a checkpoint for antiviral interferon production. EMBO J. (2026) 45:1136–74. doi: 10.1038/s44318-026-00695-2 41571995 PMC12910037

[22] Tang Y Wang X Li Z He Z Yang X Cheng X . Heparin prevents caspase-11-dependent septic lethality independent of anticoagulant properties. Immunity. (2021) 54:454–67:e6. doi: 10.1016/j.immuni.2021.01.007 33561388

[23] Liu ZS Cai H Xue W Wang M Xia T Li WJ . G3BP1 promotes DNA binding and activation of cGAS. Nat Immunol. (2019) 20:18–28. doi: 10.1038/s41590-018-0262-4 30510222 PMC8276115

[24] Tasdemir E Galluzzi L Maiuri MC Criollo A Vitale I Hangen E . Methods for assessing autophagy and autophagic cell death. Methods Mol Biol. (2008) 445:29–76. doi: 10.1007/978-1-59745-157-4_3 18425442

[25] Marwaha R Sharma M . DQ-Red BSA trafficking assay in cultured cells to assess cargo delivery to lysosomes. Bio Protoc. (2017) 7. doi: 10.21769/bioprotoc.2571 29082291 PMC5657473

[26] Pan BS Perera SA Piesvaux JA Presland JP Schroeder GK Cumming JN . An orally available non-nucleotide STING agonist with antitumor activity. Science. (2020) 369. doi: 10.1126/science.aba6098 32820094

[27] Yan N . Immune diseases associated with TREX1 and STING dysfunction. J Interferon Cytokine Res. (2017) 37:198–206. doi: 10.1089/jir.2016.0086 28475463 PMC5439420

[28] Nehar-Belaid D Hong S Marches R Chen G Bolisetty M Baisch J . Mapping systemic lupus erythematosus heterogeneity at the single-cell level. Nat Immunol. (2020) 21:1094–106. doi: 10.1038/s41590-020-0743-0 32747814 PMC7442743

[29] Chaussabel D Quinn C Shen J Patel P Glaser C Baldwin N . A modular analysis framework for blood genomics studies: Application to systemic lupus erythematosus. Immunity. (2008) 29:150–64. doi: 10.1016/j.immuni.2008.05.012 18631455 PMC2727981

[30] Crow YJ Manel N . Aicardi-Goutieres syndrome and the type I interferonopathies. Nat Rev Immunol. (2015) 15:429–40. doi: 10.1038/nri3850 26052098

[31] Niewold TB Kelly JA Flesch MH Espinoza LR Harley JB Crow MK . Association of the IRF5 risk haplotype with high serum interferon-alpha activity in systemic lupus erythematosus patients. Arthritis Rheum. (2008) 58:2481–87. doi: 10.1002/art.23613 18668568 PMC2621107

[32] Lisnevskaia L Murphy G Isenberg D . Systemic lupus erythematosus. Lancet. (2014) 384:1878–88. doi: 10.1016/s0140-6736(14)60128-8 24881804

[33] Furie R Khamashta M Merrill JT Werth VP Kalunian K Brohawn P . Anifrolumab, an anti-interferon-alpha receptor monoclonal antibody, in moderate-to-severe systemic lupus erythematosus. Arthritis Rheumatol. (2017) 69:376–86. doi: 10.1016/j.berh.2017.10.005 28130918 PMC5299497

[34] Lah TT Hawley M Rock KL Goldberg AL New Collective A . Gamma-interferon causes a selective induction of the lysosomal proteases, cathepsins B and L, in macrophages. FEBS Lett. (1995) 363:85–9. doi: 10.1016/0014-5793(95)00287-j 7729559

[35] Park B Brinkmann MM Spooner E Lee CC Kim YM Ploegh HL . Proteolytic cleavage in an endolysosomal compartment is required for activation of Toll-like receptor 9. Nat Immunol. (2008) 9:1407–14. doi: 10.1038/ni.1669 18931679 PMC2735466

[36] Ewald SE Lee BL Lau L Wickliffe KE Shi GP Chapman HA . The ectodomain of Toll-like receptor 9 is cleaved to generate a functional receptor. Nature. (2008) 456:658–62. doi: 10.1038/nature07405 18820679 PMC2596276

[37] Ewald SE Engel A Lee J Wang M Bogyo M Barton GM . Nucleic acid recognition by Toll-like receptors is coupled to stepwise processing by cathepsins and asparagine endopeptidase. J Exp Med. (2011) 208:643–51. doi: 10.1084/jem.20100682 21402738 PMC3135342

[38] Shang J Hu B Kung KK Jiang J Zhang Q Wong MK . Identification of cathepsin L as the molecular target of hydroxychloroquine with chemical proteomics. Mol Cell Proteomics. (2025) 24:101314. doi: 10.1016/j.mcpro.2025.101314 41115470 PMC12719729

