# Supplementary material for: Cathepsin L potentiates autoimmunity by inhibiting lysosome-mediated STING degradation
Source: Front Immunol. 2026 Aug 26;17:1920873. doi: 10.3389/fimmu.2026.1920873 (PMC13553787; doi:10.3389/fimmu.2026.1920873)
Supplement: Supplementary file 1 [file Presentation1.pdf]

Supplementary Material

Cathepsin L potentiates autoimmunity by inhibiting lysosome-mediated STING degradation

Jia-Qing Xing[1†], Zhi-Hao Zhang[1†], Zeng-Lin Guo[1], Hong Cai[1], Qiu-Ying Han[1], Jie Pan[1], Min-Yi Feng[1], Wen Xue[1], Ming Zhao[1], Kai Wang[1], Xin Xu[1], Tao Li[1,2], Tian Xia[1]*, Li-Ming Sun[1]*

Affiliations:

[1]Nanhu Laboratory, State Key Laboratory of Biomedical Analysis (SKLBA)[#], Beijing 100039, China.

[2]Institute of Translational Medicine, Zhejiang University, Hangzhou 310029, China

[†]These authors contributed equally: Jia-Qing Xing, Zhi-Hao Zhang.

[#]Formerly known as National Center of Biomedical Analysis.

*Correspondence: txia@ncba.ac.cn; lmsun@xmail.ncba.ac.cn

Supplementary Figure 1 CTSL deficiency impairs STING-mediated IFN signaling

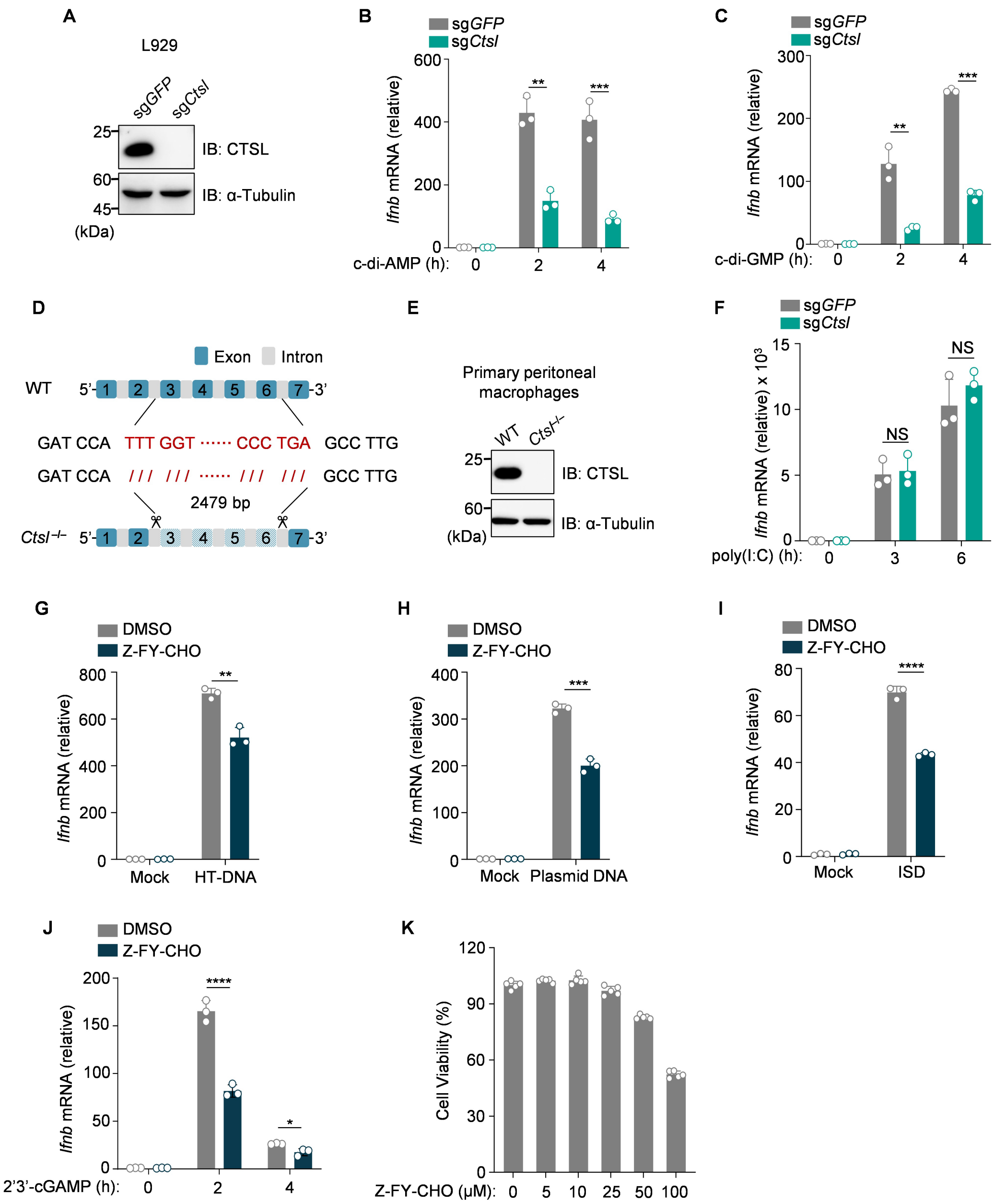

Supplementary Figure 1 CTSL deficiency impairs cGAS-STING-mediated IFN signaling.

(A) Immunoblot analysis of the indicated proteins in control and *Ctsl*-deficient L929 cells. **(B, C)** qPCR analysis of *Ifnb* mRNA expression in c-di-AMP (20 μg/ml) **(B)** and c-di-GMP (20 μg/ml) **(C)** treated L929 cells. **(D)** Schematic diagram of the deletion of *Ctsl* in WT and *Ctsl*$^{-/-}$ mouse. **(E)** Immunoblot analysis of the indicated proteins in WT and *Ctsl*$^{-/-}$ mouse PMs. **(F)** qPCR analysis of *Ifnb* mRNA expression in L929 cells transfected with poly(I:C) (1 μg/ml). **(G-J)** The effect of Z-FY-CHO on HT-DNA- **(G)**, plasmid DNA- **(H)**, ISD- **(I)** and 2'3'-cGAMP **(J)** induced *Ifnb* production in L929 cells. **(K)** MTS assay shows the cell viability of L929 cells incubated with Z-FY-CHO at indicated concentrations for 48 h. Data are shown as mean ± SD of n = 3, NS, not significant, ** p<0.01, *** p<0.001, **** p<0.0001, two-tailed unpaired Student's t-test.

Supplementary Figure 2 CTSL knockout leads to the degradation of STING

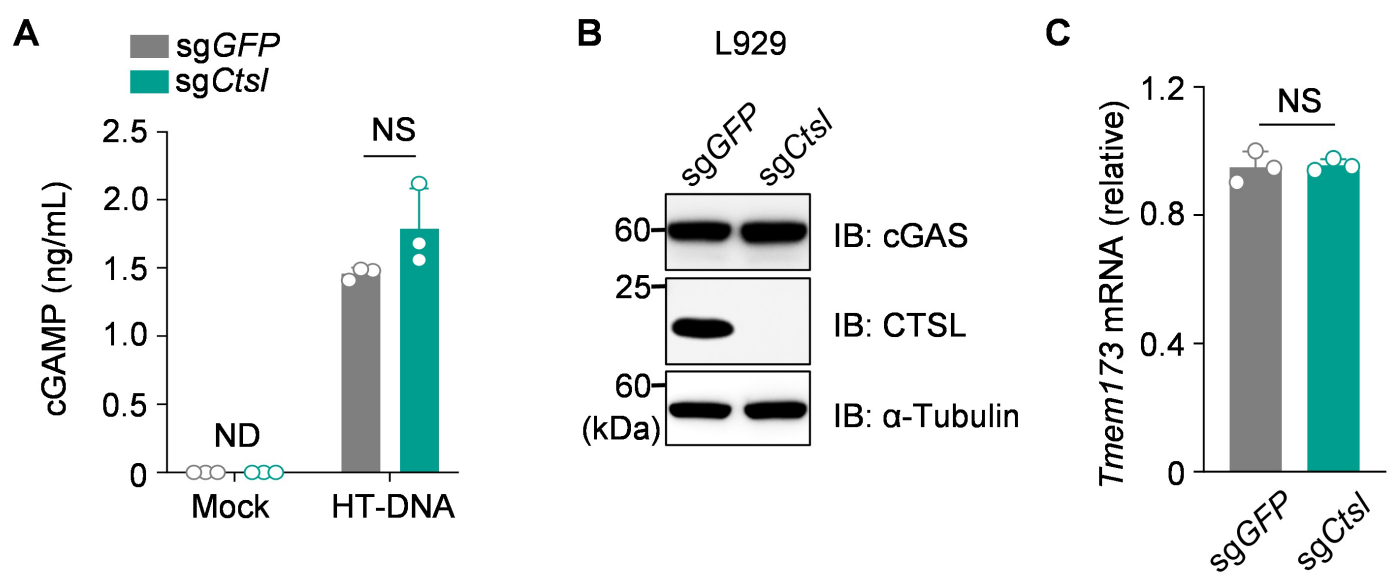

Supplementary Figure 2 CTSL knockout lead to the degradation of STING.

(A) ELISA analysis of cGAMP production of HT-DNA-treated control or *Ctsl*-deficient L929 cells. **(B)** Immunoblot analysis of the indicated proteins in control and *Ctsl*-deficient L929 cells. **(C)** qPCR analysis of basal *Tmem173* mRNA in control or *Ctsl*-deficient L929 cells. Data are shown as mean ± SD of n = 3, NS, not significant, two-tailed unpaired Student's t-test.

Supplementary Figure 3 Gating strategy of Lyso-Tracker positive cells

A

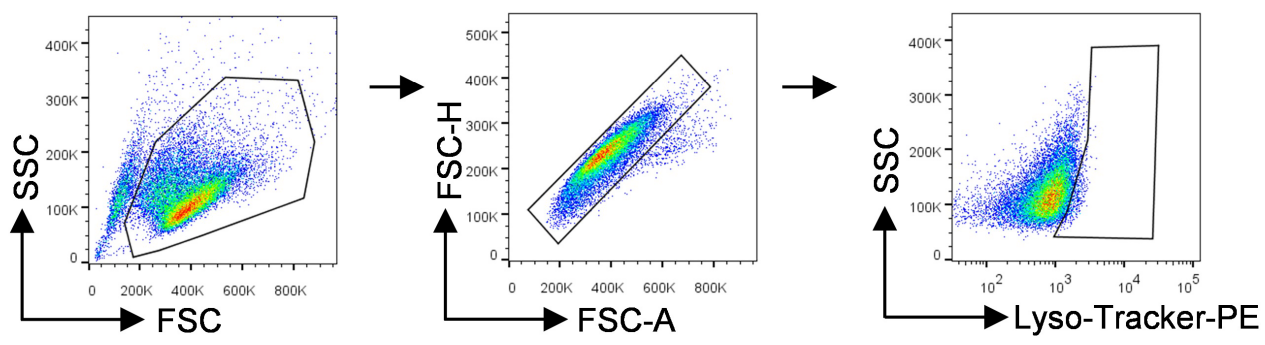

Supplementary Figure 3 Gating strategy of Lyso-Tracker positive cells.

(A) Gating scheme for Lyso-Tracker positive cells.
